# Computationally grafting an IgE epitope onto a scaffold: Implications for a pan anti-allergy vaccine design

**DOI:** 10.1016/j.csbj.2021.08.012

**Published:** 2021-08-14

**Authors:** Sari S. Sabban

**Affiliations:** a King Abdulaziz University, Faculty of Science, Department of Biological Sciences, Jeddah, Saudi Arabia

**Keywords:** Protein design, Epitope grafting, Vaccine design, Computational structural biology, Allergy, Type I hypersensitivity

## Abstract

Allergy is becoming an intensifying disease among the world population, particularly in the developed world. Once allergy develops, sufferers are permanently trapped in a hyper-immune response that makes them sensitive to innocuous substances. The immune pathway concerned with developing allergy is the Th_2_ immune pathway where the IgE antibody binds to its Fc∊RI receptor on Mast and Basophil cells. This paper discusses a protocol that could disrupt the binding between the antibody and its receptor for a potential permanent treatment. Ten proteins were computationally designed to display a human IgE motif very close in proximity to the IgE antibody’s Fc∊RI receptor’s binding site in an effort for these proteins to be used as a vaccine against our own IgE antibody. The motif of interest was the FG loop motif and it was excised and grafted onto a *Staphylococcus aureus* protein (PDB ID 1YN3), then the motif + scaffold structure had its sequence re-designed around the motif to find an amino acid sequence that would fold to the designed structure correctly. These ten computationally designed proteins showed successful folding when simulated using Rosetta’s AbinitioRelax folding simulation and the IgE epitope was clearly displayed in its native three-dimensional structure in all of them. These designed proteins have the potential to be used as a pan anti-allergy vaccine. This work employed*in silicobased methods for designing the proteins and did not include any experimental verifications.*

## Graphical abstract

**Figure:**
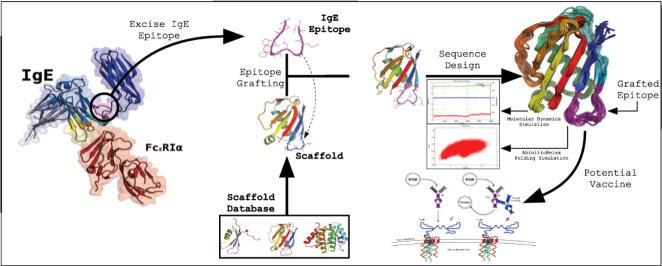

## 1 Background

Allergy was first defined by Clemens von Pirquet in 1906 when he discovered that second injections of horse serum caused a severe inflammatory reaction in some, but not all, individuals. He termed this condition Allergy, from the Greek words allos “other” and ergon “works” and therefore the allergy-causing agent was called an “allergen” [1]. In the 1960s Kimishige Ishizaka and Teruko Ishizaka demonstrated that allergic reactions are mediated by a new class of antibodies that they discovered and called immunoglobulin E [2], [3], which binds onto a receptor called the high-affinity IgE receptor (Fc∊RI) which is found on Mast and Basophil cells and comprises four chains (an α extracellular chain with two domains, an intermembrane β chain, and two intermembrane γ chains protruding into the cytoplasm).

Humans have five antibody types (IgA, IgD, IgE, IgG, and IgM). Immunoglobulin G (IgG) is the most abundant antibody type since it mainly targets viral and bacterial pathogens. Immunoglobulin E (IgE) on the other hand, is thought to be concerned with extracellular parasitic infections, where an association was found between *Schistosoma mansoni* infections and higher levels of serum IgE [4], as well as noxious toxin immunity (such as venom) [5] where it seems the immune system attacks forgein enzymes such as *Apis mellifera* (bee) phospholipase A2 (Api m 1), *Dermatophagoides pteronyssinus* (mite) peptidase (Der p 1), and *Persea americana* and (avocado) endochitinase (Pers a 1). This pathway can target innocuous substances that look like parasites or toxins but are not usually harmful such as *Olea europaea* (olive) pollen (Ole e 1), leading to a type of inflammatory reaction termed an allergic reaction, or known medically as type I hypersensitivity.

Thus, IgE antibodies are best known for their role as mediators of the allergic response, which in its most serious manifestations, causes asthma or an anaphylactic shock. Reports of an increase in the number of individuals suffering from allergic manifestations began in the second half of the last century and the incidence of allergy has now reached pandemic proportions [6]. IgE-mediated allergic responses have diverse manifestations, which range from mild to severe and can be life-threatening. Mammals including humans, dogs, and horses are known to suffer the clinical symptoms of IgE-mediated type I hypersensitivity responses. Despite extensive worldwide research efforts, no effective active therapeutic intervention strategies are currently available.

One of the perceived reasons for the continual increase in allergy incidence, especially in the developed world, is a hypothesis termed the Hygiene Hypothesis, originally formulated by Strachan [7], [8], [9], it states that a lack of exposure to infectious pathogens in early childhood, i.e. living in an environment too clean, can lead to inadequate immune system development, i.e. a shift from the Th_1_ immune response (bacteria, viruses) to that of the Th_2_ immune response (parasite, allergy), increasing the susceptibility to develop allergy. Further studies in this immunological pathway have shed light on the viability of this hypothesis and showed a correlation between tuberculosis infections in childhood and lack of allergy in adulthood [10].

Currently, the most widely used therapy against allergy is pharmacotherapy, which is a passive immunotherapeutic intervention strategy employing the use of antihistamines, corticosteroids, or epinephrine, all of which alleviate the symptoms of allergy without curing its underlying cause. The quest to treat allergy is not a new concept, it was first attempted in 1911 [11] when subcutaneous injections (subcutaneous immunotherapy or SCIT) of an allergen extract were administered in an effort to desensitise atopic patients to certain allergens. Both SCIT and SLIT work by repeated administration of the allergen in increasing doses, this is thought to prompt B and T cells to switch antibody classes from IgE to IgG reducing the symptoms of allergy as well as diminish the late-phase immune response [12]. SLIT was successful to treat certain conditions such as anaphylaxis and allergic rhinitis, while older studies showed variable success in treating asthma [13] newer studies are finding better success [12]. This protocol has remained controversial as it has the potential to sensitise patients even more, thus worsening their condition [14]. Another immunotherapy called sublingual immunotherapy (SLIT) is also being researched where allergen extracts are given to patients under their tongues [15]. The efficacy of these therapies varies greatly between individuals since doctors do not have a standardised protocol to follow, they usually develop their own protocols according to their own observations and individual successes.

**Fig. 1:**
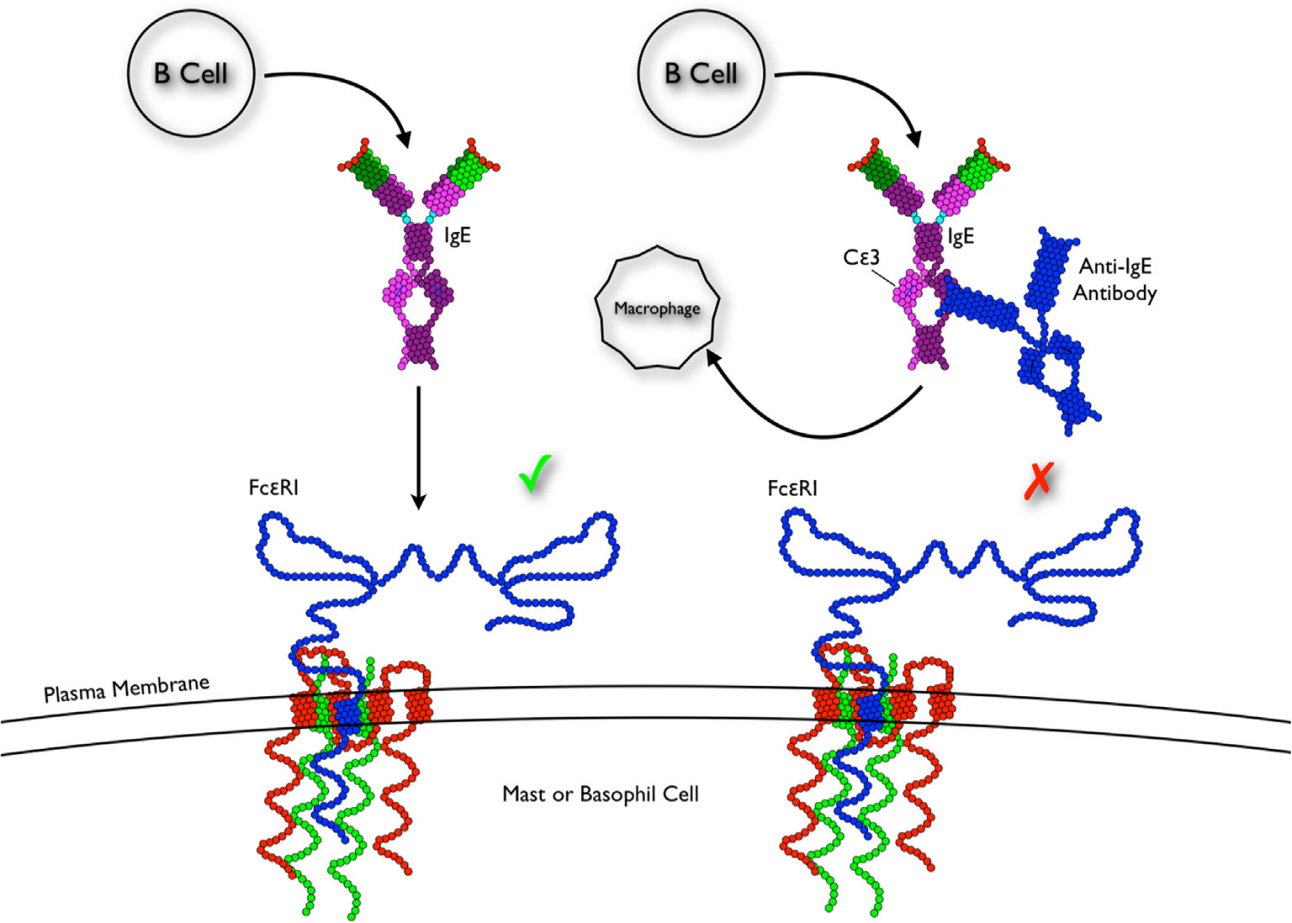
Summery of the pan-anti allergy vaccine therapy concept. Administering a vaccine that is capable of producing antibodies against the body’s own self IgE molecule, and neutralising it by preventing it from binding onto its receptor, could disrupt the entire allergy pathway and potentially curing the disease [23].

Since allergy incidence has been on the rise globally, a new form of therapy is under development. Though still a passive immunotherapeutic strategy, it employs non-anaphylactogenic antibodies which have demonstrated their capacity to treat type I hypersensitivity responses. These humanised mouse monoclonal antibodies (mAbs), of which Omalizumab [16] is best characterised, are now successful in treating severe forms of allergy but have been shown to be associated with a number of drawbacks: 1) poor effectiveness in obese patients, 2) logistics and cost, 3) treatment only reduces symptoms temporarily, hence it is a passive immunotherapeutic strategy. While Omalizumab (PDB ID 5G64) [17] binds onto the IgE antibody on a location very close to the Fc∊RI binding site and interferes with Fc∊RI and Fc∊RII (CD23) binding, the antibody 026 (PDB ID 5NQW) [18] binds onto a similar location on the IgE further away from the Fc∊RI binding site, but still manages to interfere with Fc∊RI and Fc∊RII binding. Both these strategies are passive immunisation approaches. The drawbacks of passive immunotherapy logically lead to the potential to develop new active forms of immunotherapeutic strategies, such as a vaccine that primes the immune system against its own IgE antibody, at which point the IgE is neutralised and the allergy disease is terminated. Even though current mainstream research is concentrating on the passive immunisation approach, it is believed that active immunisation is a viable form of treatment against this disease (Fig. 1). This paper is discussing a potential active immunisation strategy (a potential vaccine) that, in theory, would prompt the immune system to target a location on the IgE molecule very close to the Fc∊RI receptor binding site, with the goal of generating antibodies that would disrupt or mask the binding site itself. Thus a pan-anti allergy vaccine can be computationally designed by excising the motif (a motif on or near the IgE antibody’s receptor binding site) of interest from the IgE structure and grafting it onto a scaffold protein structure, thus displaying only the motif of interest in its original three-dimensional form without any of the surrounding native structure, allowing the immune system to target just that particular motif. Computationally grafting epitopes onto scaffold proteins was previously detailed in [19], that protocol relied mainly on the geometric similarity of the motif/scaffold complex measured by a sequence-based prediction method and utilised statistical rotamers for sequence designing the final protein. This paper on the other hand used an automated energy function-based approach from the Rosetta software, where the motif was structurally grafted (based on the backbone geometry) onto a scaffold and the final grafted structure redesigned then forward folded, the difference here is that the structures in this paper were being directed by the REF2015 energy function [20], [21], [22].

The IgE molecule was chosen as a target primarily as a continuation of the work done on Omalizumab and antibody 026, where these antibodies are passive anti-IgE immunisation strategies the question here is whether it is possible to develop a similar yet active anti-IgE immunisation strategy. Furthermore, targeting one motif on the IgE should in theory turn off the pathway and reduce the IgE blood titer, while targeting the Fc∊RI receptor might cause the immune system to attack and reduce the number of mast and basophil cells causing wider disruptions to the immune system itself.

## 2 Materials and methods

The following steps were used to generate a database of scaffold protein structures to search for a an appropriate backbone to graft the motif onto, as well as isolate the IgE motif, graft it onto the found scaffold, then design the scaffold to fold onto the designed protein structure.

### 2.1 Motif determination and excision

**Fig. 2:**
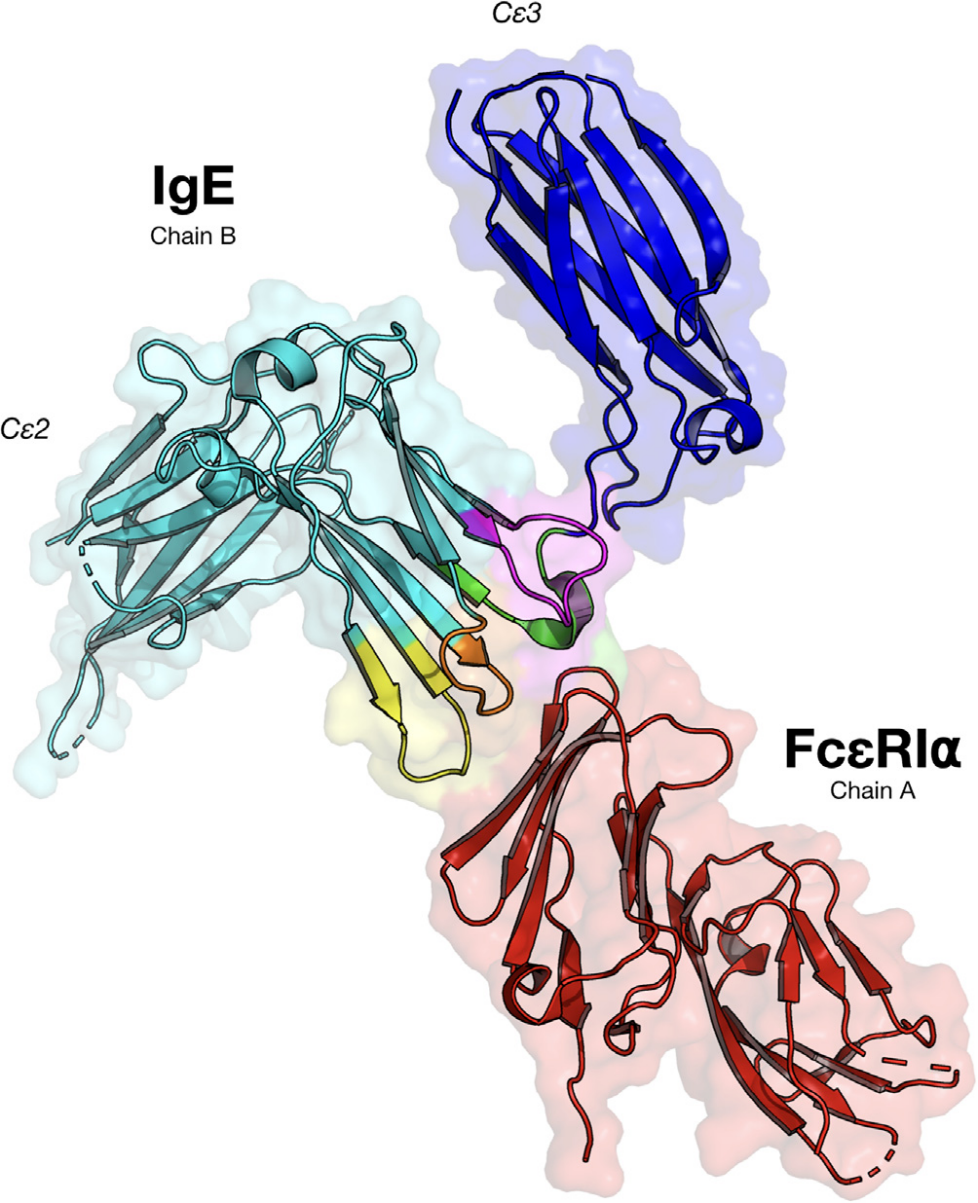
The structure of the human IgE bound to its Fc∊RIα receptor (PDB ID 2Y7Q) [24]. The colours show the different loops that are closet in proximity or forms hydrogen bonds with the receptor when bound. Purple for the FG loop, green for the R loop, orange for the BC loop, and yellow for the DE loop.

The FG loop (Fig. 2 purple colour) from the human IgE crystal structure (PDB ID 2Y7Q, chain B, amino acids 420–429 with the sequence VTHPHLPRAL) [24] was chosen due to its very close proximity to the IgE’s receptor binding site (PDB ID 2Y7Q, chain B, amino acids 331–338 with the sequence SNPRGVSA) named the R loop (Fig. 2 green colour). The FG loop motif has a ridged structure and it forms a beta sheet loop with an anchoring lysine at position 425 pointing into the core fixing its shape. Since the IgE’s receptor binding site (R loop) was not anchored in place with an amino acid pointing into the core giving it a higher degree of movement resulting it in not being well modelled in the crystal structure, hence it was not chosen (this can be clearly observed when looking at chain C where the same position is missing), furthermore, when the R loop motif was grafted it assumed multiple structures as can be seen in Fig. 2, thus the FG loop motif was chosen instead. Furthermore, the omalizumab antibody (which reduces serum IgE) binds to the cε3 region of the IgE making contact with the FG loop rather than the R loop in the crystal structure (PDB ID 5G64) [17]. The FG loop motif was isolated along with the full receptor chain (PDB ID 2Y7Q all of chain A) as separate.pdb files in preparation for grafting. In the (PDB ID 2Y7Q) crystal structure only the receptor’s extracellular α chain is modelled with its two domains. In this paper, the FG and R loops refer to the loops in the original human IgE molecule as seend in Fig. 2, while the terms FG loop motif and R loop motif refer to these loops as 3D structures and sequences that have been isolated and grafted onto scaffold proteins.

### 2.2 Scaffold database generation

The scaffold database was generated by downloading the entire protein databank using this command:

**Table:** 

rsync -rlpt -v -z --delete --port = 33444 rsync.wwpdb.org::ftp/
data/structures/divided/pdb/./PDBdatabase

Each structure was unzipped and the original zipped structures deleted to save memory space. Then, each structure with multiple chains was separated into separate.pdb files for each chain using this simple python script that uses the biopython [25] python library:

**Table:** 

**import**os
**import**Bio.PDB
io = Bio.PDB.PDBIO()
**for**TheFile**in**os.listdir(’PDBdatabase’):
TheName = TheFile.split(’.’)[0].upper()
structure = Bio.PDB.PDBParser(QUIET = True).get_structure(TheName, TheFile)
**for**chain**in**structure.get_chains():
io.set_structure(chain)
io.save(’./PDBdatabase/’+structure.get_id()+’_’+chain.get_id()+’.pdb’)

Then desired structures (sizes below 150 amino acids) were isolated using the following bash code:

**Table:** 

mkdir chosen**for**file**in**PDBdatabase/*.pdb;**do**
CHAINAnumb=‘grep ATOM $file |**awk**’{print $5 "⧹t" $6}’ | grep A |
tail -n 1 |**awk**’{print $2}’‘
CHAINBnumb=‘grep ATOM $file |**awk**’{print $5 "⧹t" $6}’ | grep B |
tail -n 1 |**awk**’{print $2}’‘
[[$CHAINBnumb = *[!0--9]* || $CHAINAnumb = *[!0--9]*]] && **continue**
AminoAcids=$((CHAINBnumb-CHAINAnumb)) echo $AminoAcids
**if**(($AminoAcids ⧹ < 150))
**then**
mv $file chosen;
**fi**
**done**

Following that step, structures were cleaned (removed any none-peptide atoms such as water, ion, and ligands) using the following Linux terminal bash command:

grep -e ATOM -e MSE -F PDBID_CHAIN.pdb > PDBID_CHAIN_clean.pdb for example:

grep -e ATOM -e MSE -F 3HZ7_A.pdb > 3HZ7_A_clean.pdb

Care must be taken to ensure that the non-canonical MSE (selenomethionine, which is used to solve crystal structures) amino acid is transferred to the new cleaned structure since it is under the HETATM heading. MSE amino acids in.pdb under the HETATM heading replaces MET (methionine) amino acids under the ATOM heading. If MSE was not imported it will result in structures with missing selenomethionine. To ensure that the structures are compatible with PyRosetta and will not crash it they are run through this script (basically just imported then exported):

**Table:** 

**import**os
**from**pyrosettaimport*
init()
os.makedirs(’cleaned’, exist_ok = True)
**for**TheFile**in**os.listdir(’chosen’):
pose = pose_from_pdb(’./chosen/{}’.**format**(TheFile))
pose.dump_pdb(’./cleaned/{}’.**format**(TheFile))

Structures that were not satisfactory were deleted. In this way the scaffold database was constructed.

### 2.3 Scaffold search and motif grafting

The desired IgE motif (the FG loop motif) with the sequence VTHPHLPRAL between positions 420 and 429 in chain B of the protein crystal structure with PDB ID 2Y7Q was isolated along with all of chain A, which was the Fc∊RI receptor’s α chain since in the 2Y7Q crystal structure only the receptor’s extracellular α chain was modelled with its two domains. A scaffold search was performed where the motif was grafted onto each structure within the scaffold database using the epitope grafting protocol [22], [21]. If there was a match within an RMSD value of 1.0 Å or less then the grafted structure (with the motif replacing the original backbone on the scaffold) was measured for its clash with the receptor (i.e: to make sure the backbone was not grafted inward or was buried within the structure). If there was no clash with the receptor structure, then the final grafted structure was exported. The code used for this step can be found in this GitHub repository.

### 2.4 Selective fixed-backbone sequence design

The final grafted structure was tested for folding (in the next section), which failed to converge into a low root-mean-square deviation (RMSD) value and a low Rosetta free energy score. Thus, to find a sequence that would allow the grafted structure to fold into the desired structure it had to be sequence designed, i.e: find a sequence that would fold into the desired structure. Initially, this was attempted manually by human-guided mutations where amino acids were mutated at strategic locations, chosen visually to fill in core voids using only amino acids that were specific to the secondary structures of the mutation site, putting into consideration their layer position calculated by each amino acid’s solvent accessible surface area (SASA) using the same calculation criteria in [26]. After several failed attempts, the RosettaDesign fixed-backbone design protocol was employed [27], [28], [29], [30], [31]. The side chains (amino acid identities) of the structure were stochastically mutated and packed using a rotamer library to find the lowest energy structure that would fold into the designed backbone. In this protocol, the REF2015 energy function weights were changed to include aa_rep 1.0, aspartimid_penalty 1.0, buried_unsatisfied_penalty 1.0, and approximate_buried_unsat_penalt 5.0, which assisted in designing an adequate sequence that fits the backbone structure and increased the energy gap between the desired fold and any other possible undesired fold. The code used for this step can be found in this GitHub repository.

### 2.5 Folding simulation

To get insight into whether or not the design process was successful, the folding of the sequence-designed-grafted-structure was simulated using the Rosetta [32] AbinitioRelax protocol [33], [34], [35], [36], [37], [38], which employed a Monte Carlo method, where the amino acid sequence is used to construct a straight primary structure, then 3-mer and 9-mer fragments were randomly inserted. The fragments were generated from the Robetta fragment server (http://robetta.bakerlab.org/fragmentsubmit.jsp) using the amino acid sequence. These fragments are backbone torsion angles of secondary structures that were statistically calculated from the amino acid sequence and they help speed up the simulation. Then the structure was randomly moved (backbone and side-chain torsion angles changed) and its free energy was calculated using the REF2015 scoring function which employs first physical principles and some statistical weights [20] using the following equation (details are explained in the original paper):

$$\Delta E_{\mathrm{total}}=\underset{i}{\sum }w_{i}E_{i}(\Theta_{i},{\mathrm{aa}}_{i})$$

After several cycles of moving and scoring the final structure was exported. This was repeated 1 million times, which results in 1 million simulated structures. These structures were then plotted on a score vs RMSD plot to show how similar they are to the originally designed structure. A successful simulation would result in a funnel-shaped plot, where the lowest scoring structures (lowest free calculated energy) result in structures close to the designed structure (low RMSD) since it is assumed that any protein structure resides within the global free energy minima. The code used for this step can be found in this GitHub repository.

## 3 Results and discussion

**Fig. 3:**
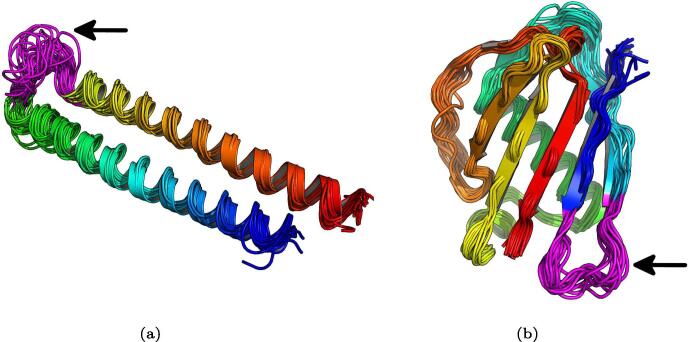
Comparison of grafting the R loop motif to grafting the FG loop motif. A: Folding simulation of the R loop motif (in purple) after successfully being grafted onto the 3Q4H protein and sequence designed, here showing a large variability of the motif backbone since it lacked an anchor (average RMSD = 1.29 Å to the natives motif). B: Folding simulation of the FG loop motif (in purple) after successfully being grafted onto the 1YN3 protein and sequence designed, here showing better motif stability (average RMSD = 0.62 Å to the natives motif). Structures rendered through PyMOL [41].

Analysis of several motif positions revealed that the R loop and the FG loop from the human IgE (PDB ID 2Y7Q) are the best candidates for a targeted vaccine due to their proximity to the binding site on the α chain of the Fc∊RI receptor (Fig. 2). After several attempts at grafting and designing the R loop motif onto a *Mycobacterium smegmatis* EsxGH protein (PDB ID 3Q4H replacing the sequence QGDTGMTY at positions 44–51), the FG loop motif appeared to be the better choice, this was due to the FG loop motif having an inward-pointing leucine, resulting in a ridged loop structure, compared with the R loop motif that had a high degree of angle freedom, which resulted in a wide range of different structures when grafted, see Fig. 3 for a comparison between the grafted and designed motif coloured in purple.

**Fig. 4:**
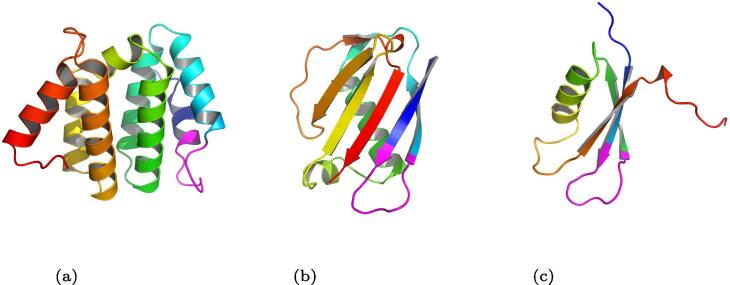
Grafting the FG loop motif onto three different scaffolds. This figure is showing three structures that successfully accepted the grafted the FG loop motif A: A domain of the human STAM1 VHS (PDB ID 3LDZ chain A) where the ATSEMNTAED sequence at positions 16–25 was replaced by the FG loop motif. B: An EAP domain protein from *Staphylococcus aureus* (PDB ID 1YN3 chain A) where the sequence ITVNGTSQNI at positions 164–173 was replaced by the FG sequence. C: The PAAB subunit of the Phenylacetate-CoA Oxygenage from *Ralstonia eutropha* (PDB ID 3EGR chain B) where the VRSKQGLEHK sequence at positions 13–22 was replaced by the FG loop motif. 1YN3 was chosen since the native structure was easily forward folded using AbinitioRelax (Fig. 5). Thus it was easier to redesign this structure and it did result is a structure with a large energy gap between the desired structure and any other potential structure.

**Fig. 5:**
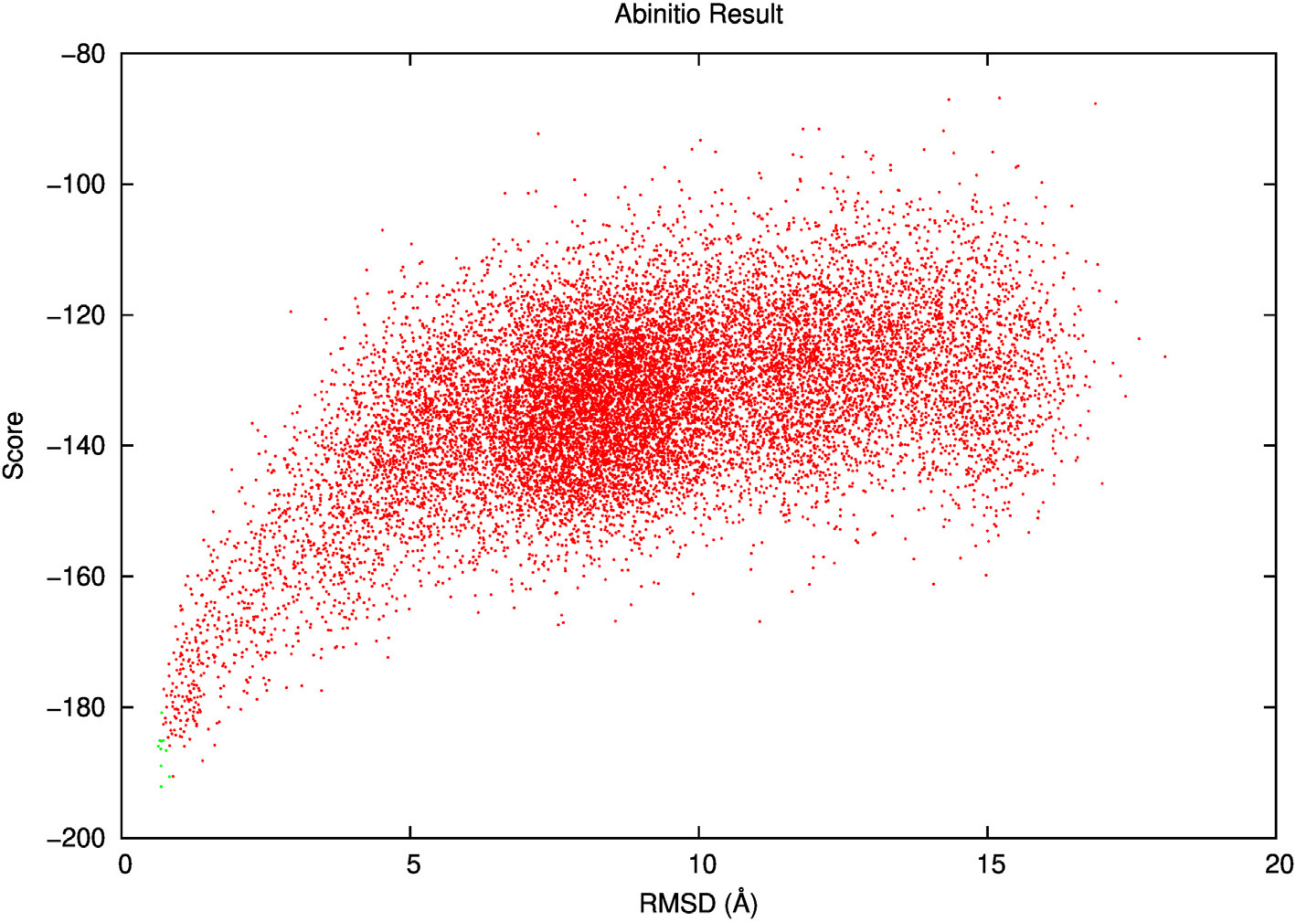
Folding simulation of the native 1YN3 protein structure. AbinitioRelax result of the native 1YN3 protein showing a successful simulation, a funnel shaped plot with the lowest simulated energy close to the predicted energy and RMSD of the structure.

**Fig. 6:**
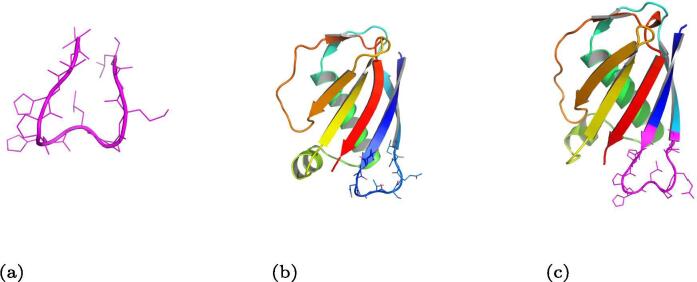
Stages of the grafting protocol. A: The native structure of the motif. B: The native structure of the *Staphylococcus aureus* EAP protein (PDB ID 1YN3) used here as a scaffold C: The final structure after the motif in purple was grafted onto the scaffold, then only the scaffold sequence designed (not the motif).

The scaffold search resulted in the FG loop motif being grafted onto a *Staphylococcus aureus* EAP protein (PDB ID 1YN3) [39] structure as well as several other structures (Fig. 4). The 1YN3 structure was chosen because it had a backbone that was easily simulated by forward folding using the AbinitioRelax protocol (Fig. 5) when tested as a control on the original wild type crystal structure. Another reason was that the 1YN3 protein is a *Staphylococcus aureus* protein which was expressed in *Escherichia coli* when it was crystallised and is highly antigenic, thus it is predicted to easily crystallise for final structural evaluation and the backbone could instill a strong immune response, which is required to develop antibodies that would bind to the IgE at a stronger affinity than the IgE binds onto its receptor. The motif was grafted between positions 164 and 173 on the 1YN3 structure replacing the sequence ITVNGTSQNI with VTHPHLPRAL (Fig. 6).

**Fig. 7:**
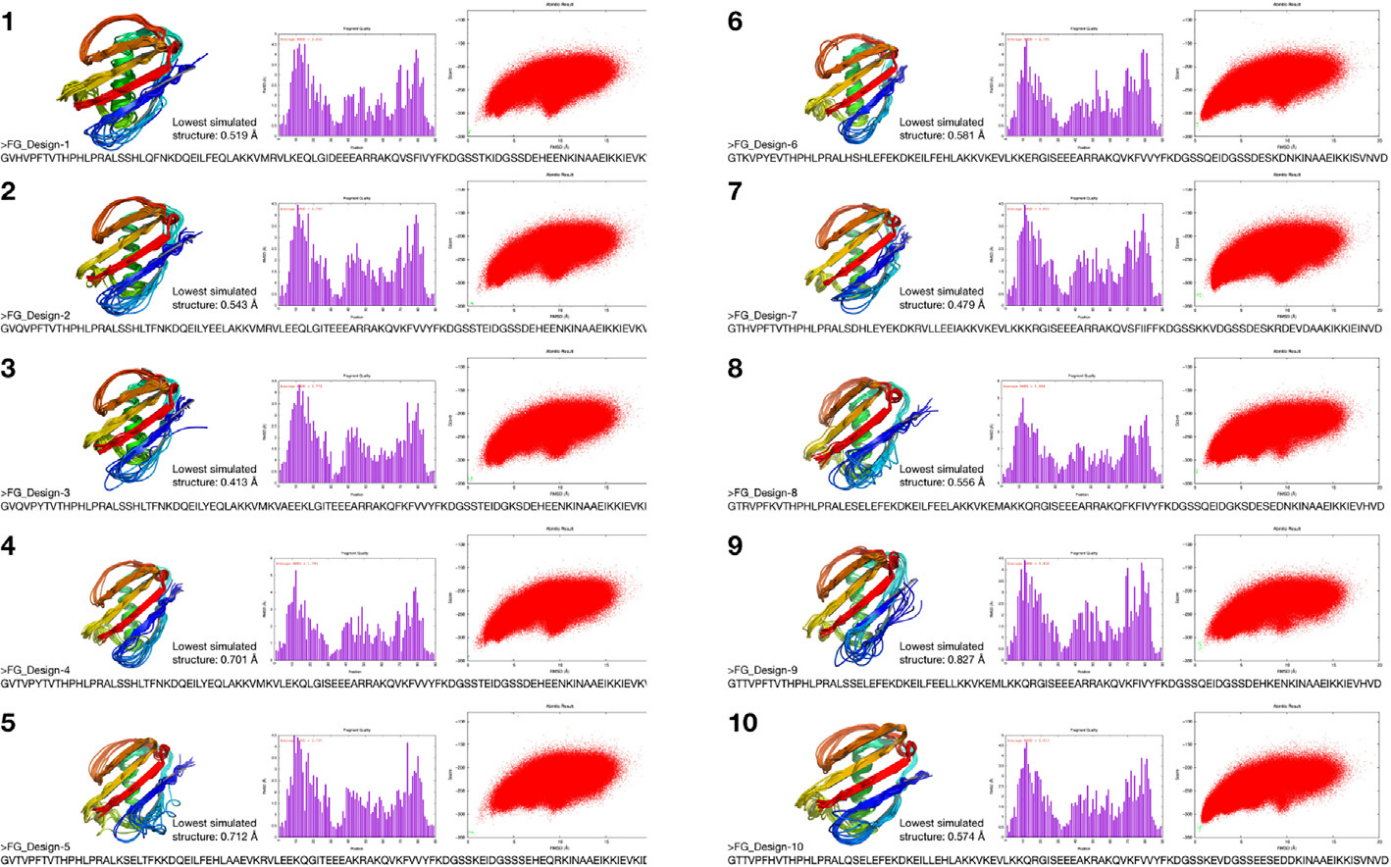
Final designed structures. Ten successfully designed structures that display the FG loop motif in its native three dimensional structure. The figure shows each designed structure (cartoon) superimposed onto the lowest energy and lowest RMSD structures from the AbinitioRelax simulation (wire) and the corresponding lowest RMSD value of the simulation, thus all structures were predicted to fold within a sub angstrom level of the designed structure giving high confidence that the proteins will have this fold when they are physically synthesised. Also showing are the FASTA sequences of each structure, the fragment quality used in each AbinitioRelax simulation, and the AbinitioRelax plot showing a successful funnel shaped plot for all structures. The green points in each folding simulation are the REF2015 (Rosetta Energy Function 2015) energy score values of the corresponding computationally designed structure after being relaxed thus indicating the lowest possible energy score for each structure and is thus used as a baseline to show were the global minima could be.

As predicted the freshly grafted structure failed a forward fold simulation using AbinitioRelax, this was due to the addition of the motif backbone and side chains severely disrupted the stability of the entire structure. To overcome this, the entire structure was sequence designed by changing and optimising the side chains (except for the motif) while fixing the backbone to stabilise the structure and accommodate the new motif backbone and side chains. At first, manual sequence design was performed, which proved fatal, then the RosettaDesign protocol was successfully used as described in the methods section. Since a failure rate exists between a successful forward fold and a successful crystal structure, the sequence design step was repeated ten times, this resulted in ten structures with the same motif and backbone but different sequences, all of which had a successful forward folding simulation (Fig. 7). This should increase the probability of synthesising a correctly folded vaccine structure since only one of these structures must pass a crystallography evaluation to be tested as a potential vaccine. If several structures do pass the crystallography evaluation, one structure can be used as a vaccine, while the others are used as a boosts.

The following are the sequences of all the structures, aligned with each other to highlight the differences:

**Table:** 

1: GVHVPFTVTHPHLPRALSSHLQFNKDQEILFEQLAKKVMRVLKEQLGIDEEEARRAKQVSFIVYFKDGSSTKIDGSSDEHEENKINAAEIKKIEVKVD		
2: GVQVPFTVTHPHLPRALSSHLTFNKDQEILYEELAKKVMRVLEEQLGITEEEARRAKQVKFVVYFKDGSSTEIDGSSDEHEENKINAAEIKKIEVKVD		
3: GVQVPYTVTHPHLPRALSSHLTFNKDQEILYEQLAKKVMKVAEEKLGITEEEARRAKQFKFVVYFKDGSSTEIDGKSDEHEENKINAAEIKKIEVKID		
4: GVTVPYTVTHPHLPRALSSHLTFNKDQEILYEQLAKKVMKVLEKQLGISEEEARRAKQVKFVVYFKDGSSTEIDGSSDEHEENKINAAEIKKIEVKVD		
5: GVTVPFTVTHPHLPRALKSELTFKKDQEILFEHLAAEVKRVLEEKQGITEEEAKRAKQVKFVVYFKDGSSKEIDGSSSEHEQRKINAAEIKKIEVKID		
6: GTKVPYEVTHPHLPRALHSHLEFEKDKEILFEHLAKKVKEVLKKERGISEEEARRAKQVKFVVYFKDGSSQEIDGSSDESKDNKINAAEIKKISVNVD		
7: GTHVPFTVTHPHLPRALSDHLEYEKDKRVLLEEIAKKVKEVLKKKRGISEEEARRAKQVSFIIFFKDGSSKKVDGSSDESKRDEVDAAKIKKIEINVD		
8: GTRVPFKVTHPHLPRALESELEFEKDKEILFEELAKKVKEMAKKQRGISEEEARRAKQFKFIVYFKDGSSQEIDGKSDESEDNKINAAEIKKIEVHVD		
9: GTTVPFTVTHPHLPRALSSELEFEKDKEILFEELLKKVKEMLKKQRGISEEEARRAKQVKFIVYFKDGSSQEIDGSSDEHKENKINAAEIKKIEVHVD		
10: GTTVPFHVTHPHLPRALQSELEFEKDKEILLEHLAKKVKEVLKKQRGISEEEAKRAKQVKFVVYFKDGSSKEVDGSSEESEDDKINAAEIKKISVNVD		
*. **: ********** ..* ::**:.:* *.: :* .: ::: ** ****:****..*:::****** ::**.*.*: :::**:****.:::*		

To analyse the structures further, all their FASTA sequences were used to predict the secondary structures of the final proteins. The following are the predicted secondary structures using PSIPRED [40] (H for helix, E for Strand, and C for Coil), *des* is for the designed structure’s secondary structures and *pre* is for predicted secondary structures from the designed structure’s amino acid FASTA sequence:

**Table:** 

Original 1YN3 scaffold crystalstructure:		
actual CEEEEEEEEECCCCCCEEEEEEECCCCEEEHHHHHHHHHHHHHHHHCCCHHHHHHCCCEEEEEEECCCCEEEEECCCCCCCCCEEEHHHEEEEEEEEC predict CCCCCEEEEECCCEEEEEEEEEECCCCCCCHHHHHHHHHHHHHHCCCCCHHHHHHCCEEEEEEEEECCCEEEEECCCCCCCCCCCCHHHCEEEEEEEC		
		
Designed structures: des 1: CEEEEEEEECCCCCCCEEEEEEECCCCEEEHHHHHHHHHHHHHHHHCCCHHHHHCCCCEEEEEEECCCCEEEEECCCCCCCCCEEEHHHEEEEEEEEC pre 1: CCCCCCEEEECCCCCCEEEEECCCCCCCCCHHHHHHHHHHHHHHHHCCCHHHHHHCCEEEEEEEEECCCEEEEECCCCCCCCCCCCHHHCEEEEEEEC		
		
des 2: CEEEEEEEECCCCCCCEEEEEEECCCCEEEHHHHHHHHHHHHHHHHCCCHHHHHCCCCEEEEEEECCCCEEEEECCCCCCCCCEEEHHHEEEEEEEEC pre 2: CCCCCCEEEECCCCEEEEEEEEECCCCCCCHHHHHHHHHHHHHHHHCCCHHHHHHCEEEEEEEEECCCCEEEEECCCCCCCCCCCCHHHCEEEEEEEC		
		
des 3: CEEEEEEEECCCCCCCEEEEEEECCCCEEEHHHHHHHHHHHHHHHHCCCHHHHHCCCCEEEEEEECCCCEEEEECCCCCCCCCEEEHHHEEEEEEEEC pre 3: CCCCCCEEEECCCCEEEEECEECCCCCCCCHHHHHHHHHHHHHHHHCCCHHHHHHCCEEEEEEEECCCCEEEEECCCCCCHHCCCCHHHCEEEEEEEC		
		
des 4: CEEEEEEEECCCCCCCEEEEEEECCCCEEEHHHHHHHHHHHHHHHHCCCHHHHHCCCCEEEEEEECCCCEEEEECCCCCCCCCEEEHHHEEEEEEEEC pre 4: CCCCCEEEEECCCCCEEEEEEEECCCCCCCHHHHHHHHHHHHHHHHCCCHHHHHHCEEEEEEEEECCCCEEEEECCCCCCHHHCCCHHHCCEEEEEEC		
		
des 5: CEEEEEEEECCCCCCCEEEEEEECCCCEEEHHHHHHHHHHHHHHHHCCCHHHHHCCCCEEEEEEECCCCEEEEECCCCCCCCCEEEHHHEEEEEEEEC pre 5: CCEECEEEECCCCCCCEEEEEECCCCCCCCHHHHHHHHHHHHHHCCCCCHHHHHHCCEEEEEEEECCCCEEEEECCCCCCHHCCCCHHHCEEEEEEEC		
		
des 6: CEEEEEEEECCCCCCCEEEEEEECCCCEEEHHHHHHHHHHHHHHHHCCCHHHHHCCCCEEEEEEECCCCEEEEECCCCCCCCCEEEHHHEEEEEEEEC pre 6: CCCCCEEEECCCCCCEEEEEEEECCCCCCCHHHHHHHHHHHHHHCCCCCHHHHHHCCEEEEEEEECCCCEEEEECCCCCCCCCCCCHHHCCEEEEEEC		
		
des 7: CEEEEEEEECCCCCCCEEEEEEECCCCEEEHHHHHHHHHHHHHHHHCCCHHHHHCCCCEEEEEEECCCCEEEEECCCCCCCCCEEEHHHEEEEEEEEC pre 7: CCCCCCEEECCCCCCCCCCCEEECCCCCCCHHHHHHHHHHHHHHCCCCCHHHHHHCEEEEEEEEECCCCEEEEECCCCCCCCCCCCHHHCCEEEEEEC		
		
des 9: CEEEEEEEECCCCCCCEEEEEEECCCCEEEHHHHHHHHHHHHHHHHCCCHHHHHCCCCEEEEEEECCCCEEEEECCCCCCCCCEEEHHHEEEEEEEEC pre 8: CCCCCCEEECCCCCCCEEEEEEECCCCCCCHHHHHHHHHHHHHHHCCCCHHHHHHHCEEEEEEEECCCCEEEEECCCCCCCCCCCCHHHCEEEEEEEC		
		
des 0: CEEEEEEEECCCCCCCEEEEEEECCCCEEEHHHHHHHHHHHHHHHHCCCHHHHHCCCCEEEEEEECCCCEEEEECCCCCCCCCEEEHHHEEEEEEEEC pre 9: CCEECCEEECCCCCCCEEEEEEECCCCCCCHHHHHHHHHHHHHHCCCCCHHHHHHCCEEEEEEEECCCCEEEEECCCCCCCCCCCCHHHCEEEEEEEC		
		
des 10: CEEEEEEEECCCCCCCEEEEEEECCCCEEEHHHHHHHHHHHHHHHHCCCHHHHHCCCCEEEEEEECCCCEEEEECCCCCCCCCEEEHHHEEEEEEEEC pre 10: CCEECCEEECCCCCCCEEEEEEECCCCCCCHHHHHHHHHHHHHHCCCCCHHHHHHCCEEEEEEEECCCCEEEEECCCCCCCCCCCCHHHCEEEEEEEC		

**Fig. 8:**
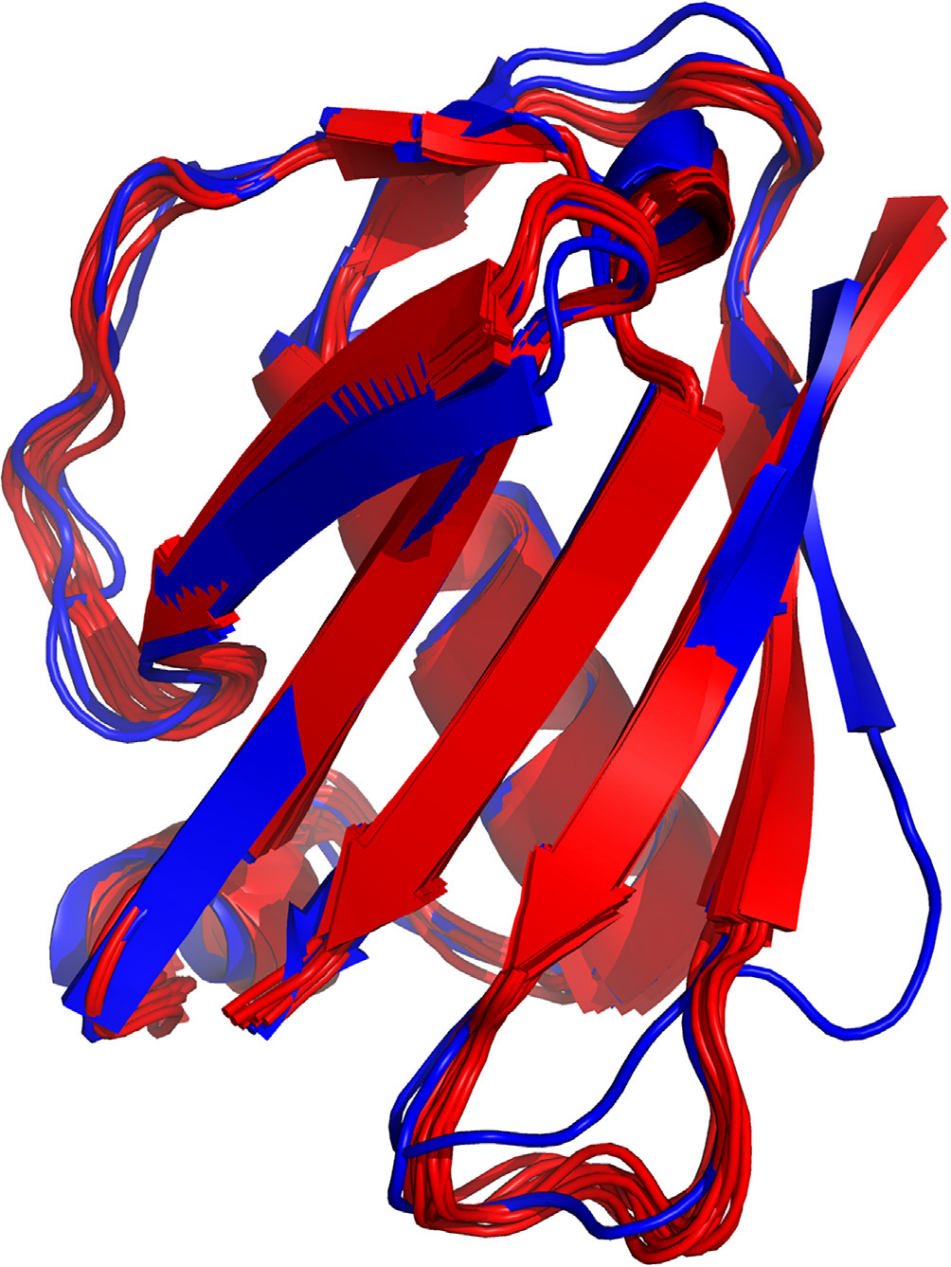
Swiss Model Predictions. The FASTA sequence of each of the designed proteins was used to predict their structure using Swiss Model [42]. Here it can be seen that the predictions from the FASTA sequence predicts similar structures to the desined structures.

Furthermore, the SWISS-MODEL tool was used to predict the structure of the designed structure from their FASTA sequence as a way to further evaluate their structures computationally. All proteins were predicted to fold as their designed structures Fig. 8.

All structures were predicted to fold within a sub angstrom level of the designed structure, giving high confidence that these will be the structures of the proteins when physically synthesised. Each structure must be crystallised and confirmed the correct fold of the protein and the motif before they can be tested on animals.

**Fig. 9:**
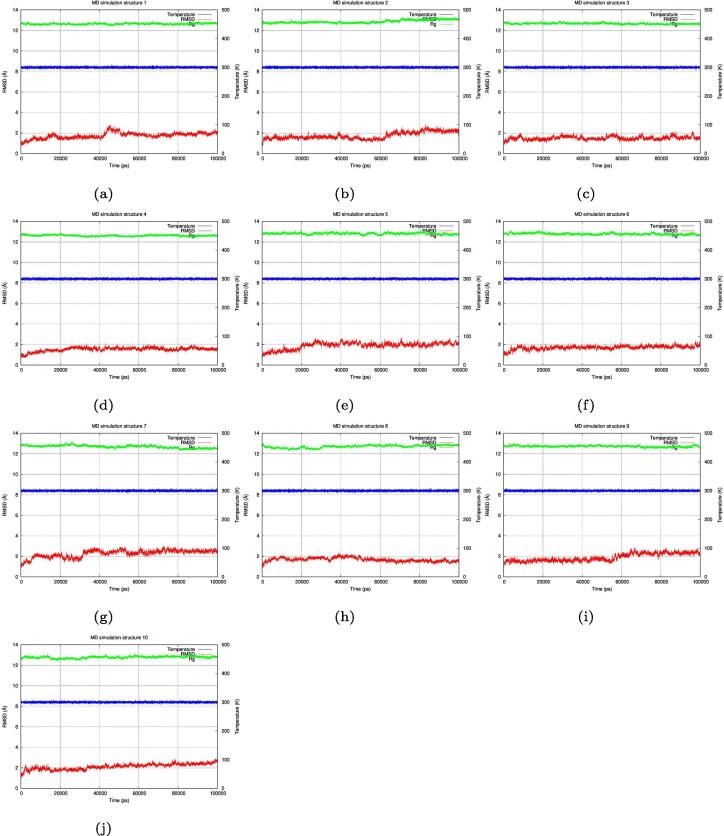
Molecular dynamics simulation at 300°K. Molecular dynamics simulation of all the 10 designed structures at 300°K (26.85 °C) for 100 ns (100,000 ps), all the structures showed a stable RMSD value (around 2 Å) where structures 3, 4, 6, and 8 showed the highest stability while structures 7, 9, and 10 showed the lowest stability. The radius of gyration for all structures was also stable (around 13 Å).

**Fig. 10:**
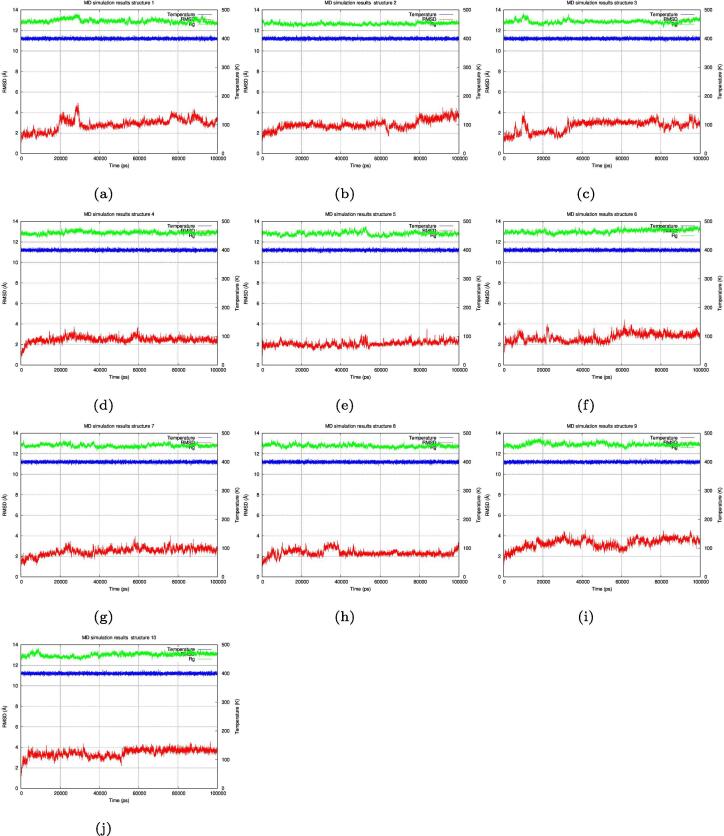
Molecular dynamics simulation at 400°K. Molecular dynamics simulation of all the 10 designed structures at 400°K (126.85 °C) for 100 ns (100,000 ps), all the structures showed less RMSD stability (fluctuating up to 4 Å and 5 Å). Structures 1, 2, 3, 4, 6, 7, 8, 9, and 10 showed low stability, reaching high RMSD values, while structures 5 showed the highest stability maintained at 2 Å on average (occasionally reaching 3 Å). The radius of gyration for all structures remained at around 13 Å.

Molecular Dynamics simulations [43], [44], [45], [46], [47], [48], [49], [50] were performed on all structures to test the stability of the folded designed structures. Initially the simulation was performed at 300°K using a 0.002 femtosecond time step for 100 ns (Fig. 9), all the structures showed an RMSD value around 2 Å. Structures 3, 4, 5, 6, and 8 showed the highest stability (RMSD values mostly less than 2 Å) and a radius of gyration value less than 13 Å. The original wild type 1YN3 radius of gyration was 12.923 Å ± 0.135 and the following list (in sequence) are of the radius of gyrations of the designed structures: [12.642 Å ± 0.075, 12.869 Å ± 0.157, 12.667 Å ± 0.074, 12.617 Å ± 0.074, 12.815 Å ± 0.093, 12.784 Å ± 0.090, 12.693 Å ± 0.141, 12.711 Å ± 0.136, 12.725 Å ± 0.084, 12.78 Å ± 0.102] all showing sub angstrom deviations from the wild type value.Structures 7, 9, and 10 showed the lowest stability (RMSD values reaching above 2 Å but less than 3 Å at the end of the simulation). The radius of gyration for all structures was around 13 Å. Then the simulation was performed at 400°K using the same parameters to test if the structures would unfold (Fig. 10). The structures showed less RMSD stability (fluctuating up to 4 Å and 5 Å). Structures 1, 2, 3, 4, 6, 7, 8, 9, and 10 showed low stability by reaching higher RMSD values than the 300°K simulation, while structure 5 showed the highest stability by maintaining an RMSD value between 2 Å and 3 Å. The radius of gyration for all structures remained at around 13 Å but with more variablity than the 300°K simulation. The original wild type 1YN3 radius of gyration was 12.929 Å ± 0.160 and the following list (in sequence) are of the radius of gyrations of the designed structures: [12.921 Å ± 0.187, 12.651 Å ± 0.119, 12.855 Å ± 0.168, 12.900 Å ± 0.139, 12.808 Å ± 0.171, 13.038 Å ± 0.177, 12.799 Å ± 0.143, 12.773 Å ± 0.142, 12.914 Å ± 0.171, 13.022 Å ± 0.177] all showing sub angstrom deviations from the wild type value.

**Fig. 11:**
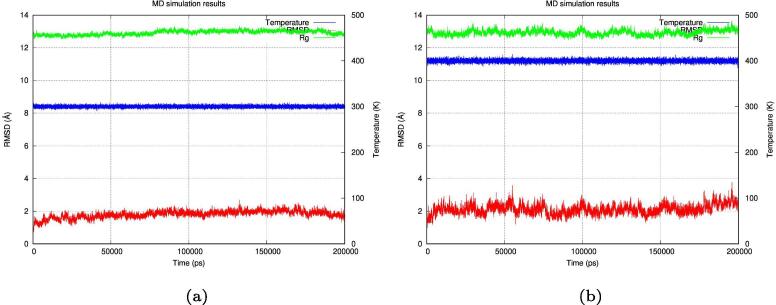
Molecular dynamics simulation of the original 1YN3 crystal structure at 300°K and 400°K. Molecular dynamics simulation of the original 1YN3 structure from the Protein Databank at 300°K (26.85 °C) and at 400°K (126.85 °C) for 100 ns (100,000 ps). A: The 300°K simulation showed a structure stable at RMSD value around 2 Å with a final RMSD value under 2 Å at the end of the simulation, it also showed a stable radius of gyration value around 13 Å at the end of the simulation. B: The 400°K simulation showed a less stable structure reaching an RMSD value of 3 Å and sometimes coming close to 4 Å with a final RMSD value above 2 Å at the end of the simulation, it also showed a less stable radius of gyration value reaching above 13 Å at the end of the simulation.

These simulations can be compared to the simulation of the original 1YN3 scaffold crystal structure from the Protein Databank (Fig. 11), where the structure was simulated at 300°K and 400°K using the same parameters. From the simulation, at 300°K the structure showed stable RMSD values (around 2 Å) with a value under 2 Å at the end of the simulation, and a radius of gyration value around 13 Å. While the simulation at 400°K the structure showed a less stable structure with less stable RMSD values (above 2 Å sometimes reaching 4 Å) with a value above 2 Å at the end of the simulation, and a less stable radius of gyration value above 13 Å. It can thus be argued that structures 3, 4, 5, 6, and 8 are the most stable structures at 300°K (26.85 °C), while structure 5 is the most stable structure at 400°K (126.85 °C).

MHC-II binding prediction was computed for the scaffold grafted FG loop motif and its surrounding amino acids (from the desgined structures) using MixMHC2pred (http://mixmhc2pred.gfellerlab.org/) [51]. Table 1 shows the top 20 peptides ranked by percentile rank with the peptide VTVPYTVTHPHLPRAL from structure 4 for HLA-II allele HLA-DQA1*02:01/DQB1*02:02 having a very good percentile rank of 0.966 (0 = best) with PYTVTHPHL being the best predicted core binding sequence indicating the possibility to elicit a CD4^+^ T cell response.

**Table 1:** MHC-II binding predictions. This table is summerising the top 20 peptides ranked by percentile rank as computed by MixMHC2pred, where Allele is the best binding HLA-II allele for that peptide, Rank is the percentile rank and the table is ordered from lowest (best binding) rank on top (0 = best, 100 = worst), Rank per Length is the percentile rank but for peptides with the same length, Best Binding Core is the predicted core binding sequence for that peptide, and Position is the predicted binding position on the binding core.

Structure	Peptide	Allele	Rank	Rank per Length	Binding Core	Position
4	VTVPYTVTHPHLPRAL	HLA-DQA1*02:01/DQB1*02:02	0.966	2.4	PYTVTHPHL	4
4	TVPYTVTHPHLPRAL	HLA-DQA1*02:01/DQB1*02:02	1.01	2.92	PYTVTHPHL	3
3	VQVPYTVTHPHLPRAL	HLA-DQA1*02:01/DQB1*02:02	1.08	2.69	PYTVTHPHL	4
3, 4	VPYTVTHPHLPRAL	HLA-DRB1*13:01	1.09	2.64	TVTHPHLPR	4
3, 4	VPYTVTHPHLPRAL	HLA-DRB1*13:01	1.09	2.64	TVTHPHLPR	4
8	KVTHPHLPRALESELE	HLA-DQA1*03:03/DQB1*03:01	1.33	3.3	PHLPRALES	5
4	GVTVPYTVTHPHLPRAL	HLA-DQA1*02:01/DQB1*02:02	1.41	2.35	PYTVTHPHL	5
10	VPFHVTHPHLPRALQ	HLA-DRB1*07:01	1.52	4.38	FHVTHPHLP	3
4	VTVPYTVTHPHLPRALS	HLA-DQA1*02:01/DQB1*02:02	1.56	2.61	PYTVTHPHL	4
3	GVQVPYTVTHPHLPRAL	HLA-DQA1*02:01/DQB1*02:02	1.57	2.63	PYTVTHPHL	5
1, 2, 5, 7, 9	VPFTVTHPHLPRAL	HLA-DRB1*13:01	1.6	3.89	TVTHPHLPR	4
5	TVPFTVTHPHLPRALK	HLA-DRB1*13:01	1.6	3.97	TVTHPHLPR	5
10	TTVPFHVTHPHLPRAL	HLA-DRB1*07:01	1.61	4	FHVTHPHLP	5
9	TVTHPHLPRALSSELE	HLA-DQA1*03:03/DQB1*03:01	1.64	4.07	PHLPRALSS	5
3	VQVPYTVTHPHLPRALS	HLA-DQA1*02:01/DQB1*02:02	1.73	2.9	PYTVTHPHL	4
5	VTVPFTVTHPHLPRAL	HLA-DQA1*02:01/DQB1*02:02	1.82	4.52	PFTVTHPHL	4
1	VHVPFTVTHPHLPRAL	HLA-DQA1*02:01/DQB1*02:02	1.91	4.74	PFTVTHPHL	4
2	VQVPFTVTHPHLPRAL	HLA-DQA1*02:01/DQB1*02:02	2	4.98	PFTVTHPHL	4
6	VTHPHLPRALHSHL	HLA-DQA1*03:03/DQB1*03:01	2.2	5.34	PHLPRALHS	4
7	THVPFTVTHPHLPRAL	HLA-DQA1*02:01/DQB1*02:02	2.2	5.46	PFTVTHPHL	4

## 4 Conclusion

This paper describes the protocol for computationally designing proteins that correctly display the three-dimensional structure of the FG loop strategic motif of the human IgE molecule. The motif was grafted onto the *Staphylococcus aureus* EAP protein (PDB ID 1YN3), which was used as a scaffold structure, then the scaffold/motif was sequence designed multiple times resulting in ten structures each with the same backbone, displaying the same motif in almost its native structure, yet each structure having a different sequence around the motif. Therefore, opening the possibility of using such protein structures as a vaccine and boosts against our own IgE to permanently shut down the allergy pathway regardless of the offending allergen (a pan-anti allergy vaccine). The resulting structures showed agreement in their final folds when simulated with the Rosetta AbinitioRelax folding algorithm, folding to sub-angstrom levels when computationally folded from their amino acid sequence’s primary structure. Nevertheless, the only definitive way to determine their realistic physical folds is to solve their structures through X-ray crystallography or NMR. Furthermore, the efficacy of the proteins in pushing the immune system into developing antibodies against our own IgE at a higher binding affinity than the IgE/Fc∊RI receptor’s binding affinity could not be computationally simulated, and thus must be tested on animals to reach a definitive answer. The script that was used to design these proteins is available at this GitHub repository, which includes an extensive README file and a video that explains how to use it.

This work performed initial testing of the hypothesis (that it is possible to graft and design a protein structure displaying a strategic human IgE epitope that can be potentially used as a vaccine and boost against human IgE) by employing *in silico* based methods for designing the proteins, but this manuscript did not include any experimental verifications. As a follow-up, experimental verifications are required to further test this hypothesis which should include synthesis and purification of all the proteins in a bacterial host (the sequence of each protein is provided in Fig. 7), testing for binding between the synthesised proteins and known anti-IgE antibodies using the enzyme-linked immunosorbent assay (ELISA), modeling of all the structures through X-ray crystallography to ensure that the FG loop motif is in the correct structure, and finally challenging animals for an immune reaction then testing their sera for binding to the proteins and the human IgE through ELISA, measuring the binding affinity of the antibodies to the proteins and human IgE through Surface Plasmon Resonance (SPR), and testing for IgE/FcεRI complex disruption through a cell-based mediator release assay [23]. The IgE/FcεRI complex binding affinity is in the order of K_D_ = ~1×10^−10^M [52]. The paper by [53] showed that it is possible to develop antibodies (such as mAb12 in that paper) with K_D_ = 1.61×10^−10^M this slightly higher affinity than the IgE/FcεRI complex reported removal of IgE molecules and IgE-bearing cells from the blood. Thus an affinity greater than K_D_ = ~1×10^−10^M would be required for a successful vaccination. On the other hand, Omalizumab’s binding affinity is in the order of K_D_ = ~3×10^−*8*^M [17] and the paper reported clinical significance, thus a lower affinity might still show some clinical significance by removing free IgE in the blood.

IgE has multiple roles in the immune system, mainly anti-parasitic, but also anti-cancer [54] roles. On the other hand, in addition to allergy IgE is also thought to have roles in autoimmune diseases, such as lupus erythematosus, bullous pemphigoid, and chronic urticaria [55]. Yet knocking out all of the IgE antibodies permanently will predictably come with side effects. These side effects are not the discussion of this paper, rather this paper is an academic answer to whether or not it is possible to design such as a vaccine. From our current understanding of the IgE molecule, permanently removing IgE will result in a weaker immune system and can be theorised to disrupt parasitic immunity as well as viral, bacterial, and possibly cancer immunity as reported by [56] where side effects of administering Omalizumab included parasitosis (Giardiasis) in one out of 19 patients in the cohort. Depending on the output of wet laboratory experiments and if the side effects were to be clearly mapped, this should give physicians the cost/benifit choice of whether to administer such a vaccine given its side effects and a patient’s disease state.

## Competing interests

The author has used these results to apply for a patent (filed by the institute) under application number US 16/988,076. The patent application is pending.

## Funding

The authors declared that no grants were involved in supporting this work.

## Availability of data and materials

The code used in this project is available at this GitHub repository which includes an extensive README file and a video that explains how to use the script.

## CRediT authorship contribution statement

**Sari S. Sabban:** Conceptualization, Data curation, Formal analysis, Funding acquisition, Investigation, Methodology, Project administration, Resources, Software, Supervision, Validation, Visualization, Writing - original draft, Writing - review & editing.

## Declaration of Competing Interest

The authors declare that they have no known competing financial interests or personal relationships that could have appeared to influence the work reported in this paper.
